# Supplementary material for: Developing a high-quality patient-centric integrated model for emergency care system in selected districts of India: An implementation research protocol (INDIA-EMS Study)
Source: PLoS One. 2025 Sep 3;20(9):e0331290. doi: 10.1371/journal.pone.0331290 (PMC12407451; doi:10.1371/journal.pone.0331290)
Supplement: S1 Table — (PDF) [file pone.0331290.s001.pdf]

Supplymentry Table 1: Formative Research Components, Stakeholders, Data Collection Techniques and Sample Size

Domains of Enquiry	Settings / stakeholders	Methods of data collection	Sample size (Indicative)
**A. Formative Research at System Level (Qualitative)**			
**Stakeholders Perspectives**	● **Health care providers (HCP) and managers:** The study participants include staff and administration of health facilities<br>● **Policy makers**: Health policy makers and health system management involved in the management and decision-making processes of the emergency care system, nodal officer trauma and emergency care, any other key personnel as identified by the State), National Health Authority, regulators, and legal advisors	Qualitative Interview FGD and IDI<br>● **Stakeholder consultation** will be done to identify the perspective of state and district<br>● **Semi-structured interviews** are also planned to be conducted with state and national level regarding financing level policy making units and programme units.<br>The study will use participatory impact pathways analysis (PIPA) & participatory social network analysis (PSNA)	● 24 HWC – 96 HCP<br>● 49 PHC- 400 HCP<br>● 16 CHC – 200 HCP<br>● 2-3 SDH/DH – 100 HCP<br>● 5-10 Private health care facilities – 100 HCPs<br>● 10 Paramedics ambulance services<br>● Policy makers –5

**Facility assessment**	• District Hospital/Medical College and Hospital<br>• Community Health Centres<br>• Primary Health Centres<br>• Health and Wellness Centres<br>• Private Hospitals	• Observation and assessment of physical infrastructure<br>• 24 hour live observations of process of care for each identified emergencies<br>• Facility Assessment Checklist (IPHS)<br>• Data on key indicators of the current state of emergency care e.g., emergency visits, types of emergencies, patient outcomes, resource availability, state of human resources, supplies, budgetary provisions abstracted from administrative data including insurance systems and through primary data collection.<br>• Call centre data and ambulance and prehospital care services data<br>• Depending on the resources available for each of the emergency conditions, the facilities will be designated as L1, L2, L3 and L4 facilities	• DH – 1<br>• Medical College - 1<br>• CHC - 16(purposive sampling, well performing and poorly performing)<br>• PHC- 49 (purposive sampling, well performing and poorly performing)<br>• HWC – 24<br>• (purposive sampling, well performing and poorly performing)<br>• Private hospitals – 10-15<br>• Ambulance system assesment -State and private.<br>• IT systems -States system

**Resource and facility mapping**	• Facility mapping will be done for all the public and tertiary care private facilities present in the district and adjoining districts..<br>• Agencies working in pre hospital care or any other aspect of the emergency care system will be identified and mapped.	• Resource Mapping using administrative records and Participatory Rapid Appraisal<br>• Geo-Spatial Maps<br>• Mapping of ambulance care services and care pathways at health facilities at different levels will also be performed	All PHC, CHC, HWC, DH, MCH, ambulance services
**Emergency care pathway**	Pathway of care	• Baseline survey<br>• Post care interviews	• Baseline survey – as per the sample size given below<br>• Post care interviews – 500 proportionately distributed to each health facility
**Competency assessment**	• Doctors delivering emergency care<br>• Nurses<br>• Paramedics	OSCE skill assessment and MCQ test for knowledge assessment (will be developed during the formative research and wil be submitted to IEC)	• 24 HWC – 96 HCP<br>• 49 PHC- 400 HCP<br>• 16 CHC – 200 HCP<br>• 2-3 SDH/DH – 100 HCP<br>• 5-10 Private health care facilities – 100 HCPs

**Administrative record keeping and Monitoring Mechanisms**	● **At SC, PHC and CHCs**<br>○ MLC Register<br>○ Emergency register<br>○ Dressing register<br>○ Referral register<br>● **At District and Medical College Hospital**)<br>○ MLC Register<br>○ Line List of Emergency patients<br>○ Referral card<br>○ Specialist register<br>○ Time records<br>○ Scanned prescription.<br>○ HMIS functioning	Checklists and observation schedule (detailed checklist will be developed during the formative research and wil be submitted to IEC)	Same as facility assessment
**B. Formative Research at Community Level (Qualitative)**			
**Individual Perspectives (KAP on care seeking)**	Patients: Individuals who have experienced an emergency condition within the study geography during the study period and have visited a health facility households, caregivers	● Patients – IDI<br>● Caregivers – IDI & FGD	● 20 IDI - patients<br>● 10 IDI – Care givers<br>● 5 FGD of care-givers
**Village level/ community Key informants**	Community representatives: Local political leaders (including Panchayats and Block Development	● FGD<br>● IDI of Sarpanch/ Block development Officer, SHG Members	● IDI - 10<br>● FGDs: 2

	Officers), village self-help groups		
**Community Health Workers involvement (KAP)**	• ASHAs<br>• AWW<br>• ANM	• FGD<br>• IDI	• ASHA – 10 IDI, 1 FGD<br>• AWW- 5 IDI, 1 FGD ANM- 2 IDI<br>(Obtained through purposive sampling)
**Stakeholder Involvement**	Potential first responders: Road safety authority (including traffic police), Ambulance drivers and paramedics community groups, Taxi and auto drivers, shopkeepers, bystanders, family members	Stakeholder Engagement Mapping	-

**C. Baseline Assessment**			
**I. Baseline survey**	A) estimate the burden of emergency medical conditions in the community<br>B) assess the health care services sought after the event thus providing information on proportion who sought care, pathway of care and referral	• Survey- Semi-structured questionnaire<br>• Multistage sampling strategy. In the first stage, population will be stratified in urban and rural areas. In rural areas, villages will be a Primary	Considering the burden of emergency conditions as 141/10000 population (Table 2), assuming a confidence level of 95% and a relative precision of 25% and design effect of one (design effect is considered as one as due to rarity of

	pathway, delay in seeking care.<br>C) expenditure incurred during care provision for the emergency medical condition and utilization of insurance services	Sampling Unit (PSU)<br>● **Villages** with a population less than 500 will be excluded from the sampling frame and villages with population of more than 5000 will be split into clusters to have an approximate population of around 2000 in each cluster. In an **Urban area**, census enumeration blocks will be a PSU. Total 120 PSUs will be randomly selected (60 each from urban and rural areas) and 100 households will be randomly selected from each PSU from the line list available with the village level health functionary.<br>● All the individuals in the selected household will be included for the purpose of data collection.	outcome the events are less likely to be clustered), the required sample size is 4292. Considering a non-response rate of 20% the sample size comes out to be 5365 which is further rounded off to 6000.
