# Supplementary material for: Developing a high-quality patient-centric integrated model for emergency care system in selected districts of India: An implementation research protocol (INDIA-EMS Study)
Source: PLoS One. 2025 Sep 3;20(9):e0331290. doi: 10.1371/journal.pone.0331290 (PMC12407451; doi:10.1371/journal.pone.0331290)
Supplement: S2 Table — (PDF) [file pone.0331290.s002.pdf]

Supplementary Table 2: Determinant framework used for implementing high quality emergency care

Note: The symbols “+" and “-" serve to indicate the “valence” of the determinants, or whether we anticipate the determinant will be a facilitator (+), a barrier (-), or have a mixed impact (+/-) on the implementation of the intervention

CFIR framework	**Characteristics**
Intervention	Ambulance Services: Processes, policies, and protocols.<br>First Responder Protocol: Establish a standard operating procedure.<br>ECS Facility Protocols: Implement systems for recognition, triage, resuscitation, and referral.<br>Facility Organization: Adjust physical layout and internal structure.<br>Essential Tools: Prioritize equipment, technologies, and telemedicine.<br>Establish systems for seamless interaction across ECS levels<br>Public Private partnership for emergency care
Inner Setting	Emergency room and ambulance infrastructure, human resource, and facilities<br>Hospital administration.(+/-)<br>Communication and coordination across the health delivery system and departments and amongst facilities. (-)<br>Shared positive openness to change, incentives and disincentives among emergency care teams(+/-)<br>Strategies in place for supporting the process of change to integrated high quality ECS(-)
Outer Setting	SDG, WHO Emergency Care systems framework (+)<br>Good Samaritan Law to all emergencies, Operational guidelines of emergency care at Primary, Community and district hospital(+)<br>NITI Ayog study, Evolving regulations on Allied health care bill 2021(+)<br>Partnership with IT teams, PPP model(-)<br>Alternative health systems(-)<br>Budgetary allocation for emergency care, ambulance services, facility level care.<br>Supply chain, human resource management (-)<br>Social-cultural-economic context of the communities the ECS system aims to serve.<br>Patient level barriers for care seeking.(-)<br>Insurance system for emergency care e.g., for EM care PMJAY(-)

Characteristic of individual	*Care providers*<br>Level of Understanding of Integrated High quality ECS(+/-)<br>Confidence, skills, resources and opportunities for training(+)<br>Readiness and Openness to learning new skills and adapting new ways of working(+)<br>Individual motivation and sense of belonging to the organization (-)<br>Resilience and experience of emergency care providers(-)<br>Emotional response, Peer influence and workload of Emergency care providers(-)<br>*Patients*<br>Sociocultural contrast of patients seeking emergency care (-)<br>Confidence in emergency care system (-)<br>Patient satisfaction, patient engagement, and Health literacy (-)
Process	Planning<br>Engaging<br>Executing<br>Reflecting and evaluating
