# Supplementary material for: Developing a high-quality patient-centric integrated model for emergency care system in selected districts of India: An implementation research protocol (INDIA-EMS Study)
Source: PLoS One. 2025 Sep 3;20(9):e0331290. doi: 10.1371/journal.pone.0331290 (PMC12407451; doi:10.1371/journal.pone.0331290)
Supplement: S3 Table — (PDF) [file pone.0331290.s003.pdf]

Supplementary Table No 3: Tracer indicators for quality of emergency care system

Emergency Care System Preparedness and Coverage Indicators

Emergency Care System Preparedness and Coverage Indicators					
**S.No**	**Domain/Subdomain/ Indicator**	**Numerator**	**Denominator**	**Means of Assessment**	**Remarks**
**1**	**Emergency Health Care Preparedness Indicators**				
**1.1**	**Prehospital and transit care**				
1	Proportion of health facilities having a fully equipped functional ambulance	Number of health facilities having an ambulance	Number of health facilities assessed	Facility Assessment	Fully equipped functional ambulance for different level of health facility will be defined during the formative phase based on expert consultation
2	Proportion of ambulance personnel trained in "at scene and transit" management of common emergencies	Number of ambulance personnel trained in "at scene and transit" management of common emergencies	Number of ambulance personnel working in the district	District level training data	
**3**	Proportion of lay first responders trained in "at scene" management of common emergencies	Number of lay first responders trained in "at scene and transit" management of common emergencies	Number of lay first responders targeted to be trained	District level training data	Number of lay first responders (eg. auto, taxi and bus drivers, shopkeepers, civil volunteers, police personnel etc) to be trained will be decided based on expert consensus and

					local consultation.
**1.2**	**Hospital Care**				
**1.2.1**	**Personnel**				
4	Proportion of health facilities having round the clock availability of complete emergency care team trained in management of medical emergencies/trauma/poisoning	Number of health facilities having round the clock availability of complete emergency care team trained in management of medical emergencies/trauma/poisoning	Number of health facilities assessed	Facility Assessment	A complete emergency care team will be defined for each level of health care facility through expert consultations and local consultations during the formative phase
**1.2.2**	**Medicine and Equipment**				
5	Percentage health facilities having availability of key/essential equipment required to provide definitive care for specified emergency condition	Number of health facilities having availability of key/essential equipment required to provide definitive care for specified emergency condition	Number of health facilities assessed	Facility Assessment	Key/essential equipment list required for providing complete emergency care will be developed for different level of health care facility to be developed based on existing evidence and expert

					consultation during the formative phase
6	Percentage health facilities having adequate availability of key/essential medicines and consumables required to provide definitive care for specified emergency condition	Number of health facilities having availability of key/essential medicines and consumables required to provide definitive care for specified emergency condition	Number of health facilities assessed	Facility Assessment	Key/essential equipment list required for providing complete emergency care will be developed for different level of health care facility to be developed based on existing evidence and expert consultation during the formative phase
**1.2.3**	**Infrastructure**				
7	Percentage health facilities having an infrastructure readiness score above a desirable level	Number of health facilities having an infrastructure readiness score above a desirable level	Number of health facilities assessed	Facility Assessment	This indicator and the desirable score developed during the formative research.
**1.2.4**	**Process**				

8	Proportion of health facilities providing care as per Standard Treatment Workflow (STW) and Standard Operating Procedure (SOP) as per the level of care for management of emergency medical condition	Number of health facilities providing care as per Standard Treatment Workflow (STW) and Standard OperatingProcedure (SOP) as per the level of care for management of emergency medical condition	Number of health facilities assessed	Facility Assessmen t	
**1.2.5**	**Governance and leadership**				
9	Proportion of health facilities conducting regular mortality audit of emergency medical condition	Number of health facilities conducting regular mortality audit of emergency medical condition	Number of health facilities assessed	Facility Assessmen t	
10	Proportion of health facilities doing complete reporting of indicators on emergency care in HMIS	Number of health facilities doing complete reporting of indicators on emergency care in HMIS	Number of health facilities assessed	Facility Assessmen t	
11	Proportion of health facilities utilizing RKS funds for emergency care	Number of health facilities utilizing RKS funds for emergency care	Number of health facilities assessed	Facility Assessmen t	
**2**	**Emergency Health Care Coverage Indicators**				
1	Proportion of patients who sought care for any emergency medical condition	Number of patients who had a emergency medical condition and sought any kind of care	Number of patients who had any emergency medical condition	Population Survey	Emergency Medical Condition to be defined based on expert consensus and study definition
2	Proportion of patients who used ambulance services to reach health facility for a	Number of patients who used ambulance services to reach health facility	Number of patients who had any emergency	Population survey	

	emergency medical condition		medical condition		
3	Proportion of patients who were provided definitive care for the specified emergency medical condition	Number of patients who had a emergency medical condition and sought care at an appropriate health facility	Number of patients who had any emergency medical condition	Population Survey/ Post care interview	Definitive care to be defined as per STW
4	Proportion of patients who were provided definitive care for the specified emergency medical condition at an appropriate health facility in a time sensitive manner	Number of patients who were provided care at an appropriate health facility in a time sensitive manner	Number of patients who had emergency medical condition and sought any kind of care	Population Survey/ Post care interview	Time sensitivity to be defined as per disease condition and decided based on expert consultation and available evidence during formative phase
5	Proportion of patients who utilized PMJAY/state government insurance schemes during care for emergency medical condition	Number of patients who utilized PMJAY/state government insurance schemes during care for emergency medical condition	Number of patients who had any emergency medical condition and sought any kind of care	Population Survey/ Post care interview	
6	Proportion of patients who had catastrophic expenditure during care for emergency medical condition	Number of patients who had a catastrophic expenditure	Number of patients who had any emergency medical condition and sought any kind of care	Population Survey/Post care interview	Catastrophic expenditure to be defined

Coverage will be disaggregated for different emergency medical conditions
