# Supplementary material for: Developing a high-quality patient-centric integrated model for emergency care system in selected districts of India: An implementation research protocol (INDIA-EMS Study)
Source: PLoS One. 2025 Sep 3;20(9):e0331290. doi: 10.1371/journal.pone.0331290 (PMC12407451; doi:10.1371/journal.pone.0331290)
Supplement: S4 Table — (PDF) [file pone.0331290.s004.pdf]

**Supp. Table No 4: Process indicators**

| Desirable indicators | | | | | | | |
|---|---|---|---|---|---|---|---|
| S.NO. | Domain/Subdomain/Indicator | Numerator | Denominator | Means of Assessment | Level of Assessment (Only for health facilities) | Frequency of assessment | Remarks |
| | **Service Access/Coverage/Utilization/quality Indicator/Output** | | | | | | |
| 2.1 | Pre hospital and transit care | | | | | | |
| 2 | Proportion who used free transport (ambulance services) to reach health facility for a emergency medical condition | Number who used free transport (ambulance services) to reach health facility | Number who had any emergency medical condition | Population survey | | | |
| 2.1.1 | Time from symptom onset to first medical contact(FMC) | Median time | - | Patient interview | At all levels | 3 monthly continous | |
| 2.2 | Hospital care | | | | | | |
| 2.2.1 | % Patients UP-Triaged* within 1hr | Total no of patients up-Triaged in ED (up-triage eg green to yellow/red, yellow to Red) | Total No. of patients triaged | Hospital health records | All levels | 3 monthly continuos | |
| 2.2.2 | Door to triage time | Median time 6-20 consecutive patients' data shift/ day/week Random At | | Live observation | All levels | 3 monthly continuos | |

| | | | | | | | |
|---|---|---|---|---|---|---|---|
| | | different duty time | | | | | |
| 2.2.3 | Door to Needle /Door to groin puncture (Av. Time) (STEMI,Stroke) | Median time | - | Live obervations and hospital health records | All levels | 3 monthly continuos | |
| 2.2.4 | % Patients thrombolyzed in Emergency | Total Patients thrombolyzed in with in window period in ED | Total No. of patients with Ischemic stroke/STEMI | Live obervations and hospital health records | All levels | 3 monthly continous | |
| 2.2.5 | Door to CT (Av. Time) (Stroke & Trauma) | Median time | - | Live obervations and hospital health records | All levels | 3 monthly continuos | |
| 2.2.6 | Time to administration of appropriate treatment (e.g.oral or IV glucose,Inotropes,antibiotics,oxygen therapy for ARI,antidotes or ASV) | Median time | - | Live obervations and hospital health records | All levels | 3 monthly continous | |
| 2.2.7 | Door to OT time (Trauma,PPH) | Median time | - | Live obervations and hospital health records | All levels | 3 monthly continous | |
| 2.2.8 | Door to delivery time (Preeclampsia) | Median time | - | Live obervations and hospital | All levels | 3 monthly continous | |

| | | | | | | | |
|---|---|---|---|---|---|---|---|
| | | | | health records | | | |
| | Proportion of patients who received TXA within first 8 hours | Numberof patients who received Tranexamic acid within 8 hours | Total number of patients with PPH or Blunt trauma | Hospital health records | Alll levels | 3 monthly continou s | |
| 2.2. 9 | Proportion of patients who received(bood or blood products ,Oxytoxin,methergin,postgr aladins) on arrival to the FMC | Number of patients who received the approriate drug | Total number of patents who who presented with PPH | Hospital Helath records | All levels | 3 monthly continou s | |
| 2.2. 10 | Proportion of patients who received,antihypertensives, Magnesum sulphate) on arrival to the FMC | Number of patients who received the appropriate drug | Total number of patents who who presented with preeclam psia | Hospital Health records | All levels | 3 monthly continou s | |
| District level | | | | | | | |
| | Awarness activities | Number of training sessions or awareness activities | - | District level meetings | | Baseline and end line | |
| | Number of collaborations or partnerships with other organisation or private sector health facilities in each district | Number of collaboration s or partnerships with other organizations ,private sector health facilities | - | District level meetings | | Baseline and end line | |

| | | | | | | | |
|---|---|---|---|---|---|---|---|
| | Number of inter-district collaborations or knowledge-sharing activities | Number of inter-district collaboration s or knowledge-sharing activities | - | District level meetings | | Baseline and end line | |
| 3 | **Outcome Indicator** | | | | | | |
| 3.1 | Health outcome | | | | | | |
| | Length of hospital stay(adult and neonatal) | Median time | - | Hospital records | All levels | 6 monthly | |
| | In-hospital mortality | Mortality occuring during the hospital stay for the emergency condition | Number of patients who had an emergenc y medical condition and visited the health facility | Hospital record | All levels | 6 momthl y | |
