# Supplementary material for: Developing a high-quality patient-centric integrated model for emergency care system in selected districts of India: An implementation research protocol (INDIA-EMS Study)
Source: PLoS One. 2025 Sep 3;20(9):e0331290. doi: 10.1371/journal.pone.0331290 (PMC12407451; doi:10.1371/journal.pone.0331290)
Supplement: S5 Table — (PDF) [file pone.0331290.s005.pdf]

Supp. Table No.5: Evaluation of India EMS Model

Domains of Enquiry	Settings / stakeholders	Methods of data collection	Sample size (Indicative)
**A. Concurrent evaluation (Quarterly)**			
**Stakeholders Perspectives**	**Health care providers** (HCP) and managers: The study participants include staff and administration of health facilities<br><br>**Policy makers:** Health policy makers and health system management involved in the management and decision-making processes of the emergency care system, nodal officer trauma and emergency care, any other key personnel as identified by the State), National Health Authority, regulators, and legal advisors	Qualitative Interview FGD and IDI<br>● **Stakeholder consultation** will be done to identify the perspective of state and district<br>● **Semi-structured interviews** are also planned to be conducted with state and national level regarding financing level policy making units and programme units.<br>The study will use participatory impact pathways analysis (PIPA) & participatory social network analysis (PSNA)	● 24 HWC – 96 HCP<br>● 49 PHC- 400 HCP<br>● 16 CHC – 200 HCP<br>● 2-3 SDH/DH – 100 HCP<br>● 5-10 Private health care facilities – 100 HCPs<br>● 10 Paramedics ambulance services<br>● Policy makers – 5

**Facility assessment**	• District Hospital/Medical College and Hospital<br>• Community Health Centres<br>• Primary Health Centres<br>• Health and Wellness Centres<br>• Private Hospitals	• Observation and assessment of physical infrastructure<br>• 24 hour live observations of process of care for each identified emergencies<br>• Facility Assessment Checklist (IPHS)<br>• Data on key indicators of the current state of emergency care e.g., emergency visits, types of emergencies, patient outcomes, resource availability, state of human resources, supplies, budgetary provisions abstracted from administrative data including insurance systems and through primary data collection.<br>• Call centre data and ambulance and prehospital care services data<br>• Depending on the resources available for each of the emergency conditions, the facilities will be designated as L1, L2, L3 and L4 facilities	• DH – 1<br>• Medical College - 1<br>• CHC - 16(purposive sampling, well performing and poorly performing)<br>• PHC- 49 (purposive sampling, well performing and poorly performing)<br>• HWC – 24<br>• (purposive sampling, well performing and poorly performing)<br>• Private hospitals – 10-15<br>• Ambulance system assesment -State and private.<br>• IT systems - States system

**Emergency care pathway/Health care seeking/Cost of care**	Pathway of care/health seeking behaviour/cost in accessing care	Post care interviews	Post care interviews – 500 proportionately distributed to each health facility
**Competency assessment**	● Doctors delivering emergency care<br>● Nurses<br>● Paramedics	OSCE skill assessment and MCQ test for knowledge assessment	● 24 HWC – 96 HCP<br>● 49 PHC- 400 HCP<br>● 16 CHC – 200 HCP<br>● 2-3 SDH/DH – 100 HCP<br>● 5-10 Private health care facilities – 100 HCPs
**Administrative record keeping and Monitoring Mechanisms**	● **At SC, PHC and CHCs**<br>○ MLC Register<br>○ Emergency register<br>○ Dressing register<br>○ Referral register<br>● **At District and Medical College Hospital**)<br>○ MLC Register<br>○ Line List of Emergency patients<br>○ Referral card	Checklists and observation schedule (detailed checklist will be developed during the formative research and wil be submitted to IEC)	Same as facility assessment

	○ Specialist register<br>○ Time records<br>○ Scanned prescription.<br>○ HMIS functioning		

**B.End evaluation**			
**Endline survey**	● estimate the burden of emergency medical conditions in the community<br>● assess the health care services sought after the event thus providing information on proportion who sought care, pathway of care and referral pathway, delay in seeking care.<br>● expenditure incurred during	Similar to baseline survey	Endline survey will be conducted after the completion of the implementation phase. Objective of the endline survey will be to estimate the increase in population coverage as compared to the baseline. The estimate of population coverage (i.e. reaching the appropriate health facility within a specified time frame as per the condition; this time frame will be decided during the formative research) obtained during the baseline survey will be used to calculate the sample size for the endline survey. Thus, the sample size for the endline survey will be

	care provision for the emergency medical condition and utilization of insurance services.		calculated after the analysis of results of baseline survey.
