# Supplementary figures and images for: Developing a high-quality patient-centric integrated model for emergency care system in selected districts of India: An implementation research protocol (INDIA-EMS Study)
Source: PLoS One. 2025 Sep 3;20(9):e0331290. doi: 10.1371/journal.pone.0331290 (PMC12407451; doi:10.1371/journal.pone.0331290)

# 1    Supplementary Figure 1: Project Management Organogram

Project Management Organogram

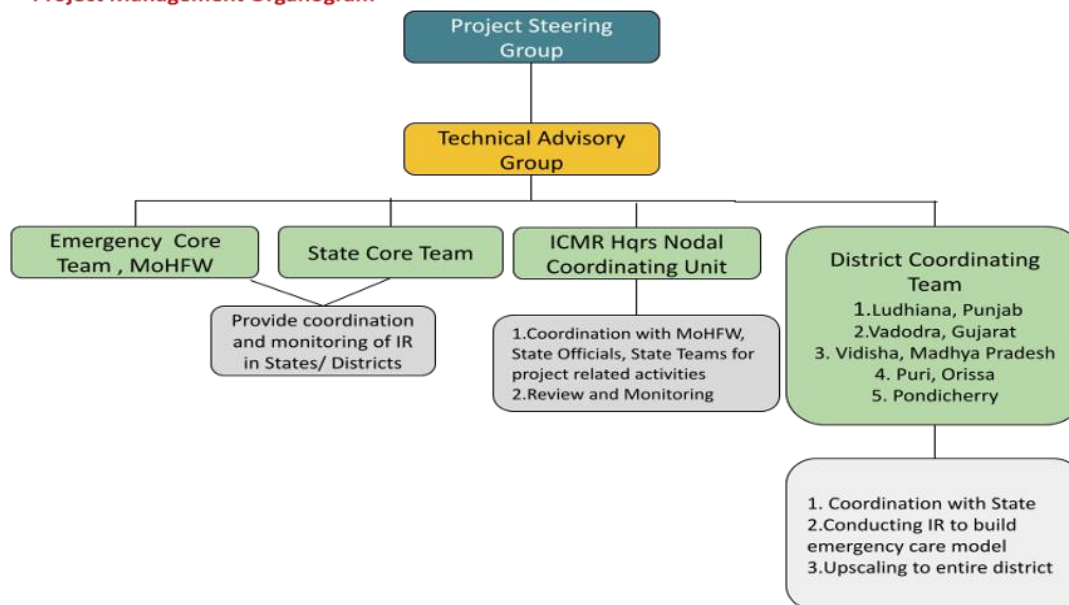

2  
3

Supplement: S1 Fig — (PDF) [file pone.0331290.s006.pdf]
